# The recording of mental health consultations by patients: clinical, ethical and legal considerations

**DOI:** 10.1192/bjb.2021.89

**Published:** 2022-06

**Authors:** Thomas Hewson, Seri Abraham, Nathan Randles, Adeola Akinola, Richard Cliff, Paul Byrne, Roshelle Ramkisson

**Affiliations:** 1 Health Education North West School of Psychiatry, UK; 2 Pennine Care NHS Foundation Trust, UK; 3 Manchester Metropolitan University, UK; 4 University of Manchester, UK; 5 University of Bolton, UK

**Keywords:** Digital health, recording consultations, service users, psychiatry and law, ethics

## Abstract

The topic of patients recording healthcare consultations has been previously debated in the literature, but little consideration has been given to the risks and benefits of such recordings in the context of mental health assessments and treatment. This issue is of growing importance given the increasing use of technology in healthcare and the recent increase in online healthcare services, largely accelerated by the COVID-19 pandemic. We discuss the clinical, ethical and legal considerations relevant to audio or visual recordings of mental health consultations by patients, with reference to existing UK guidance and the inclusion of a patient's perspective.

## Why do patients record healthcare consultations?

Recording consultations is increasingly accessible for patients as smartphones and electronic devices with recording capabilities have become ubiquitous in modern society. The integration of these devices into healthcare has been accelerated by the COVID-19 pandemic, which prompted a global upsurge in the use of telemedicine.^1^ Many digital platforms that are used for online consultations, including Zoom and Microsoft Teams, have in-built recording functions.

Several reasons have been suggested for why patients record healthcare consultations. These include enhancing their understanding of healthcare information, replaying clinical encounters, sharing these with others and for therapeutic purposes.^2^ Some patients hold digital copies of consultations to evidence the care received and to support any future complains or litigation;^2^ this may particularly be the case if patients have had prior negative healthcare experiences or lost trust in healthcare providers. For some patients, digital recordings provide a sense of ownership over their health information.^2^

## Advice from professional bodies

The General Medical Council (GMC) recommends that doctors make arrangements, wherever practical, to support patients in understanding and retaining health information; such support is described to include accommodating a patient's wish to record relevant conversations.^3^ They clearly state that doctors must obtain patients’ consent to conduct visual or audio recordings of them.^4^ However, the GMC currently does not provide formal guidance on the management of other circumstances in which patients record healthcare interactions. For example, should clinicians encourage patients to record them for reasons other than understanding and retaining information?

The Medical Defence Union (MDU), Medical Protection Society (MPS) and British Medical Association (BMA) advise that patients do not require the doctor's permission to record their healthcare consultations.^5–7^ This is because information disclosed during a consultation is confidential to the patient, not the doctor, and a recording is therefore not a breach of confidentiality in itself. Similarly, where recordings are made entirely for personal reasons, they are unlikely to engage the Data Protection Act. The MPS further advises that a doctor's duty of care should prevail over any reluctance to engage in filming by patients.^6^

Although patients can record healthcare consultations, it is less clear how the recorded material can be utilised. The MPS declares that, since ‘the content of the recording is confidential to the patient, not the doctor’ this means that ‘the patient can do what they wish with it’.^6^ However, the BMA advises that healthcare staff can request the removal of recorded materials posted online;^7^ furthermore, refusal of this request could result in litigation owing to misuse of the professional's private information. Depending on the nature of the posting, it could potentially violate the Protection from Harassment Act 1997, the Malicious Communications Act 1988 or the Communications Act 2003.^7^

Interestingly, none of the above organisations specifically refer to the mental health or mental capacity of the patient making the recording, despite the unique considerations this brings.

## The Mental Health Act

The Mental Health Act Code of Practice applies to patients detained under the Mental Health Act 1983. It states that ‘when patients are admitted, staff should assess the risk and appropriateness of patients having access to mobile phones and other electronic devices and this should be detailed in the patient's care plan’.^8^ It does not directly comment on the use of devices for recording healthcare encounters; nonetheless, it seems reasonable to apply the principles of the above statement to this situation. The terms ‘risk’ and ‘appropriateness’ suggest that professionals should consider the safety of healthcare consultations being recorded, and the suitability of the context, content and intended use(s) of any recording. These factors will vary according to individual patients and situations, demonstrating the need for a person-centred approach. If valid clinical reasons exist for denying access to electronic devices under the Mental Health Act, professionals should consider whether these extend to the recording of consultations. In the same way that clinicians note restrictions on access to personal items, they should document any reasons for preventing a patient from recording healthcare interactions. This is important for medico-legal purposes and to alert other professionals of identified risks.

## Clinical benefits of patients recording mental health consultations

The recording of healthcare consultations could help patients to remember vital information, such as self-help strategies, medication advice and suicide safety plans. This is particularly useful in psychiatry, given that concentration and attention are impaired in various mental disorders. If the patient is acutely agitated or distressed during the consultation, this could further limit their recall of conversations. Viewing the healthcare encounter when feeling more relaxed could improve a patient's adherence to medical recommendations. This could also empower patients and make them feel more involved in their care by allowing greater time for information processing.

Recordings make it easier for patients, especially those with poor recall, to inform family members about their health. This could further involve families in decision-making processes in the interests of providing holistic care. The previous recording of healthcare consultations could additionally aid decision-making processes for patients who lack mental capacity. For example, they could demonstrate a person's previously expressed wishes and values, which the team could refer to when determining the patient's best interests. This could therefore promote autonomy as an ethical principle for patients with mental illness.

Recorded consultations also potentially offer more accurate, detailed and undisputable accounts of healthcare interactions than those that are formally documented, especially for lengthy clinical encounters where clinicians must summarise vast amounts of information.^6^ Consultations recorded over time may help patients to chart their progress and response to care.

## Risks of patients recording mental health consultations

Despite the above benefits, there are several risks of recording consultations. First, this could potentially restrict the quality and quantity of information gathered throughout doctor–patient interactions. Patients may be less likely to disclose sensitive information, particularly if they intend to share the recording with others. This could influence their diagnosis and treatment, while also indirectly affecting the risks to the patient and to others. For example, a patient may withhold details of thoughts to harm family members if they are sharing the recording with these persons, limiting the validity of clinical risk assessment. Similarly, the doctor may less freely ask probing questions that expose a patient's vulnerability if they are aware of the recording being widely distributed. For these reasons, clinicians should discuss with patients which aspects of healthcare consultations they wish to record, the purpose(s) of the recording and whether this could affect their engagement or ability to provide honest information. Ideally, patients and clinicians should reach a mutually agreeable decision and work together to mitigate any potential impact on psychiatric assessment.

Some patients may lack the mental capacity to decide whether they wish to record healthcare consultations and how to use the recorded information. In such circumstances, patients could act without understanding the benefits and risks associated with their intended use(s) of the recording. This could result in harm to the patient and/or them making a decision that they later regret when they regain mental capacity. For example, a patient with mania may report reckless spending and display disinhibited behaviour during their consultation, but impulsively decide to record this and post the content online. The patient may be incapable of understanding and appraising the consequences of doing this, including heightening their risk of financial abuse and vulnerability. Furthermore, they may not recognise that they are demonstrating symptoms of mental illness, and this lack of insight could result in the unintended sharing of confidential health information. In this situation, the person would seemingly lack the mental capacity to record their healthcare encounter; consequently, the healthcare professional would have a professional and legal duty to act in their best interests. Healthcare professionals must remember that mental capacity is assumed until proven otherwise, and unwise decisions do not equate to the loss of mental capacity.^9^

Clinicians should be mindful of the content of healthcare consultations and patients’ reactions to this. Discussing sensitive topics such as suicidal thoughts, self-harm and abuse can evoke strong emotions and distress. Any intense negative emotions encountered by the patient could be re-experienced on viewing recorded consultations; in the absence of appropriate support, this could trigger acute distress and heighten the individual's risk to themselves in that present moment. Clinicians should advise patients accordingly of these risks and agree an appropriate safety plan to address them.

Social media sites are increasingly popular in modern society and some patients may post their recorded consultations on such platforms. This could result in both positive and negative comments from the public and their health information being shared beyond the original intended audience. Sharing of clips with partial information may also be misleading without providing a fuller picture of the relevant context. Clinicians should consider discussing these risks and benefits with patients, including how public reactions to private health information could affect their mental state.

## Risks to other patients and persons

Healthcare professionals must protect the safety, dignity and privacy of all patients. A common concern is that healthcare recordings may include the voice, image or details of other patients in the vicinity of the recording. This could potentially breach their privacy rights under Article 8 of the European Convention on Human Rights.^7,10^ The increased use of single bedrooms in mental health units lessens this risk; however, in-patient mental health wards are often louder than other environments and professionals should consider whether other patients are visible or audible in the background. In such circumstances, it seems best practice that the patient is offered an alternative environment for the consultation to be recorded in. If this is not feasible, the recording may need to be prevented to preserve the confidentiality and privacy of other patients. The proximity of colleagues to the recording should also be considered to protect their privacy and to avoid indirectly compromising patient confidentiality, such as by capturing a colleague's discussions relating to others.

The content of healthcare conversations can include details of third parties whose confidentiality should be protected.^11^ For this reason, when patients request access to their medical records, content relating to external persons is usually omitted before granting access.^11^ A similar process should apply to recorded consultations, with the doctor ensuring that the recorded material does not breach the confidentiality of others.

## Further ethical and legal considerations

Owing to the nature of mental illness, some patients lack the mental capacity to make decisions regarding their care. For example, approximately 40–60% of psychiatric in-patients have been estimated to lack capacity regarding treatment decisions.^12,13^ This means that recorded consultations may not accurately reflect a patient's desires and opinions, especially if these change throughout the course of the person's illness or if treatment is being provided against their wishes (under the principles of the Mental Capacity Act or the Mental Health Act). These factors should be considered when interpreting prior recordings made by patients.

## Covert recording

Studies have estimated that 26–40% of healthcare recordings by patients are made covertly.^14^ Reasons for this behaviour include distrust in the healthcare system, lack of knowledge regarding policies for ‘open’ recording and fear of recordings being prevented by clinicians.^14–16^ Covert recordings have been used in disciplinary proceedings by the GMC,^5,17^ although the BMA highlights that most recordings support the actions of doctors.^7^ To reduce covert methods, some authors have suggested that clinicians should encourage patients to visibly record their healthcare interactions.^14,15^ This could build trust, encourage shared decision-making and promote an open and honest culture within organisations. This also provides an opportunity for patients and clinicians to work together to maximise the benefits and reduce the risks of any recording, while ensuring that important non-verbal interactions are captured in any media. The practice of clinicians encouraging healthcare recordings likely requires organisational support and a clear organisational framework to support and govern this activity.

## Recordings made by carers or relatives

For some patients, their carers or relatives may attend their healthcare appointments and record consultations on their behalf. All patients who have mental capacity can refuse the recording of their health information by others, but for those without mental capacity, clinicians must consider whether any recording is in the person's best interests. This is particularly relevant in child and adolescent mental health settings, where parents are commonly involved in their children's healthcare. In the UK, children aged 13 years and above are typically deemed to have the mental capacity to access personal health records and accept or refuse parental access to these; however, there is no strict age threshold, and some children achieve mental capacity earlier than others.^18,19^ Children with the relevant mental capacity should be permitted to record their consultations and to give or deny their parents permission to do so. The healthcare professional must also consider any safeguarding concerns or relationship dynamics that could influence third party recordings and their clinical assessment. For example, children could less freely report difficulties at home if their parents are filming healthcare encounters. For patients with neurodegenerative conditions or chronic mental illness, early discussions about their healthcare preferences and other's involvement in their care could aid decision-making about recording consultations if and when mental capacity is reduced in the future. A potential benefit of recording healthcare appointments by parents or carers is that this can provide an easily accessible record and evidence of access to care when attending multiagency meetings with education and social care agencies, especially when neglect is a concern.

## Patient perspective

The following gives a patient's (N.R.'s) view on the subject.

Having spent considerable time in psychiatric and therapeutic appointments as a patient, I was initially shocked when learning of the lack of clear national guidance regarding patients’ recording of appointments in mental health settings. This lack of guidance leaves the patient vulnerable to breaches in confidentiality, potentially in cases where mental capacity is lost for the patient, and they inadvertently disclose potentially embarrassing information in public forums. We must consider the potential humiliation that service users could feel when errors occur with the use of such recordings. At the same time, recordings could provide a sense of security to patients and give them greater confidence in their care.

Ideally, there would be an independent method or platform that manages and stores recordings of healthcare consultations. This way, they could be used for the benefit of patient care and/or for medico-legal purposes, while protecting patients from some of the risks.

## Summary

Clinical services must adapt to accommodate evolving patient preferences and work collaboratively with patients to ensure that health information is appropriately and safely stored and shared with others. The recording of healthcare consultations offers several benefits to patients; however, it may also sometimes risk their privacy, safety and dignity or that of others. We recommend the establishment of clear national guidelines regarding the recording of mental health consultations. These guidelines are needed to protect both patients and professionals and are urgently required, given the increasing use of teleconsultations in mental healthcare. Such guidelines would need to acknowledge the broad range of settings and circumstances in which consultations can be recorded, including in-patient wards, home visits, community settings and online. Particular consideration needs to be given to specialist groups such as children and adolescents, patients with intellectual disabilities and persons with cognitive impairment. Furthermore, the views of numerous stakeholders must be considered, including patients, carers, multidisciplinary team members, and legal and ethical experts.

## About the authors

**Thomas Hewson**, BMBS, BMedSci (Hons), is an Academic Clinical Fellow in psychiatry with Health Education North West School of Psychiatry, UK. **Seri Abraham**, MBBS, MRCPsych, MSc, is a consultant psychiatrist with Pennine Care NHS Foundation Trust, and honorary senior lecturer at Manchester Metropolitan University, UK. **Nathan Randles** is Participation Lead with Healthy Young Minds, Pennine Care NHS Foundation Trust, UK, and a mental health service user. **Adeola Akinola**, MBChB, MRCPsych, PGDip, LLM, FHEA, is a consultant psychiatrist with Pennine Care NHS Foundation Trust and a lecturer in ethics and law at the University of Manchester, UK. **Richard Cliff**, LLB (Hons), is a trust solicitor with Pennine Care NHS Foundation Trust, UK. **Paul Byrne** is Head of Information Governance with Pennine Care NHS Foundation Trust, UK. **Roshelle Ramkisson**, MBBS, FRCPsych, MSc, PGDip, is a consultant child and adolescent psychiatrist at Pennine Care NHS Foundation Trust, honorary senior lecturer (teaching) at the University of Manchester, and senior lecturer and deputy director of the Institute of Psychiatry at the University of Bolton, UK.
